# Abdominal Wounds

**Published:** 1916-05

**Authors:** 


**Abdominal Wounds,** Sejournet, Le Prog. Med., Feb. 20, presents the following table of military experience:

| | Cases. | Cured. | Died |
|---|---|---|---|
| Penetrating wounds without visceral lesions | 11 | 8 | 3 |
| Small Intestine 1-4 perforations | 18 | 8 | 10 |
| " " more than 4 perforations | 14 | 4 | 10 |
| " " severance or tearing | 22 | 6 | 16 |
| Large Intestine | 21 | 9 | 12 |
| Large and Small Intestine combined | 22 | 6 | 16 |
| Wounds of Intestine and other viscera— | | | |
| Liver | 9 | 2 | 7 |
| Spleen | 3 | 0 | 3 |
| Bladder | 6 | 1 | 5 |
| Great blood vessels | 2 | 0 | 2 |
| Several other viscera | 4 | 0 | 4 |
| Wounds of viscera, intestine escaping— | | | |
| Liver | 12 | 9 | 3 |
| Spleen | 6 | 1 | 5 |
| Liver and other organs | 9 | 3 | 6 |
| Various wounds, kidney, bladder, great vessels | 8 | 1 | 7 |

